# Design of multi-row parallel-transmit coil arrays for enhanced SAR efficiency with deep brain electrodes at 3T: an electromagnetic simulation study

**DOI:** 10.1007/s10334-024-01212-4

**Published:** 2024-11-14

**Authors:** Nejat Karadeniz, Joseph V. Hajnal, Özlem Ipek

**Affiliations:** 1 School of Biomedical Engineering and Imaging Science, King’s College London, 3rd Floor Lambeth Wing, St Thomas’ Hospital, Westminster Bridge Road, London, SE1 7EH UK; 2 Centre for the Developing Brain, King’s College London, London, UK

**Keywords:** Implant safety, Electromagnetic simulation, Radiofrequency coils, Deep brain stimulation, Parallel transmit

## Abstract

**Objective:**

Tissue heating near the implanted deep brain stimulation (DBS) during magnetic resonance imaging (MRI) poses a significant safety constraint. This study aimed to evaluate the performance of parallel transmit (pTx) head transmit radiofrequency (RF) coils in DBS patients, with a focus on excitation fidelity under specific absorption rate (SAR) control for brain imaging at 3T MRI.

**Materials and methods:**

We employed electromagnetic simulations to assess different coil configurations, including multi-row pTx coils of 16–24 channels arranged in 1, 2, and 3 rows, and compared these to a circularly polarised and pTx birdcage coil using a realistic human model without and with DBS leads and electrodes.

**Results:**

Two- and three-row pTx coils with overlapping loop elements exhibited similar performance, which was superior in excitation homogeneity and local SAR compared to the single-row coil and the birdcage coil both without and with DBS.

**Discussion:**

These findings suggest that multi-row coils can enhance the safety and efficacy of MRI in patients with DBS devices, so potentially improving imaging performance in this expanding patient population. There was a minimal difference in performance between the 2 and 3-row coils, favouring the simpler, lower channel count design for practical implementation.

**Supplementary Information:**

The online version contains supplementary material available at 10.1007/s10334-024-01212-4.

## Introduction

Deep brain stimulation (DBS) is a well-established treatment technique for Parkinson’s disease, essential tremor, and primary dystonia and has been recommended for the treatment of psychiatric disorders [1–4]. To date, over 244,000 patients worldwide have undergone DBS surgery for a variety of neurological and non-neurological conditions [5]. Some clinicians favour the use of peri-procedure magnetic resonance imaging (MRI) for verification of implant placement. Subjects with electrodes in situ may later require MRI for a variety of reasons. Unfortunately, there is a potential risk of tissue heating due to the interaction between the radio frequency field (RF) and DBS device [6–8]. When a transmit coil generates required RF magnetic fields (B_1_^+^), electric fields are also generated, and these can induce currents to flow along the length of the DBS leads, which generally extend out through the brain and skull and then, subcutaneously, down to an implanted pulse generator (IPG) at the chest wall. Such induced currents can cause heating in conductive tissue, particularly near the lead ends [9–11].

Rezai et al. [7] conducted in vitro experiments to demonstrate the heating caused by MRI procedures on patients with implanted DBS leads. They selected either the implanted IPG or the tips of the DBS leads as the landmark position for MRI. The experiments showed that temperature increases at the lead tips ranged from 2.5 to 25.3 °C when MRI was performed using a transmit/receive body coil. A transmit/receive head coil resulted in temperature changes ranging from 2.53 to 7.1 °C. Both cases could potentially cause physical harm to the patient.

Therefore, MRI scanning after DBS surgery is constrained by safety rules to a 1.5T static field strength and root mean square (RMS) of B_1_^+^ fields under 2 μT, or in the absence of an RMS setting, a specific absorption rate (SAR) less than 0.1 W/kg, which is 30 times below the FDA limit for imaging without conductive implants [12]. These restrictions can lead to reduced image quality, and there may be challenges in maintaining a low SAR in clinical practice. Furthermore, the interaction between MRI radiofrequency fields and DBS generates artefacts [13], impeding the detection of bleeding-related abnormalities and electrode misalignments. Although 3T is considered the clinical optimum for neuro MRI, this field strength is currently contraindicated for DBS patients.

The RF-induced currents in DBS devices can be reduced by increasing the resistivity of the implant [14, 15] and adding inductances or capacitances along the conductive lengths of the implant [16]. Alternative techniques can be utilised to reduce RF-induced currents, such as modifying the DBS lead design [17] or materials [18] and modifying the RF excitation. Overall et al. [19] utilised the reserved RF polarisation to identify potential implant heating, which could then be used to mitigate heating risks. Eryaman et al. [20] noted that linear birdcage coils have a zero-electric field plane, which can be exploited to enhance safety if the implants can be regarded as lying in a single plane that can be co-aligned to prevent heating. Golestanirad et al. [21] employed a similar approach to Eryaman’s using a rotating birdcage coil mechanism to steer the position of the zero-electric field plane.

A possible general solution to prevent tissue heating around DBS without modifying the DBS hardware itself is parallel transmission (PTx) technology. PTx systems have multiple independent transmit channels, enabling the use of RF transmit coils with multiple elements, each of which can be driven with an independently controlled amplitude and phase. Eryaman et al. [22], McElchearan et al. [23–25], and Guerin et al. [26] performed electromagnetic (EM) simulations of anatomically realistic digital human phantoms with DBS present for 4, 8, or 16-element pTx coils. They demonstrated that pTx can mitigate the RF MRI safety problem. These simulations were conducted on simplified, unilateral, and bilateral DBS lead trajectories obtained by computed tomography (CT) data segmentation. Eryaman et al. [22] and McElchearan et al. [25] use a homogeneous human shape phantom, while Guerin et al. [26] use heterogeneous patient models with more realistic tissue properties. These studies have demonstrated that local SAR can be reduced by more than 94% compared to body-coil excitation while maintaining comparable B_1_^+^ homogeneity and global SAR.

Evidence for mitigation of implant heating using pTx has also been achieved through methods other than simulation-based approaches. For instance, Etezadi-Amoli et al. [27] constructed a 4-channel planar pTx system and demonstrated that transmit field combinations may be generated while limiting the current caused in a guidewire equipped with a current sensor. Gudino et al. [28] utilised a setup similar to that of Etezadi-Amoli et al. They used a coil-driving function derived from an electromagnetic simulation of the implant to determine the complex-valued coil element drive weights (“shims”) that minimise the induced temperature at the tip of the guidewire. It has been demonstrated that an 8-channel pTx coil outperforms a 4-channel pTx coil in suppressing unwanted SAR hotspots induced by DBS leads [25].

The findings indicate that pTx systems have the potential to mitigate excessive SAR caused by the presence of medical implants in patients undergoing MRI. Notably, it has been demonstrated that multi-row coil arrays provide superior control over excessive tissue heating [26] and enhance transmit efficiency and homogeneity, particularly in the longitudinal (head-foot) direction at ultra-high fields [29–31].

In this work, we revisit the potential benefits of multi-row arrays as demonstrated by Guerin et al. [26], by evaluating the performance of various head transmit RF coils at 3T for DBS patients. Our research delves into the coil design aspect, encompassing matching, tuning, and coupling for entire pTx coils to more accurately reflect the complex interactions present in actual multi-row coil arrays. Our calculations and optimizations extended beyond the tip of the DBS electrode, which was the approach in Ref. [26], to cover the entire region of interest for a comprehensive SAR assessment. The study includes a conventional birdcage structure for reference and compares it with arrays of loop elements arranged in one, two, and three rows. We focussed on excitation fidelity under SAR control, both in the absence and presence of bilateral DBS electrodes, for standard brain imaging targets.

## Methods

### Electromagnetic modelling

**Fig. 1:**
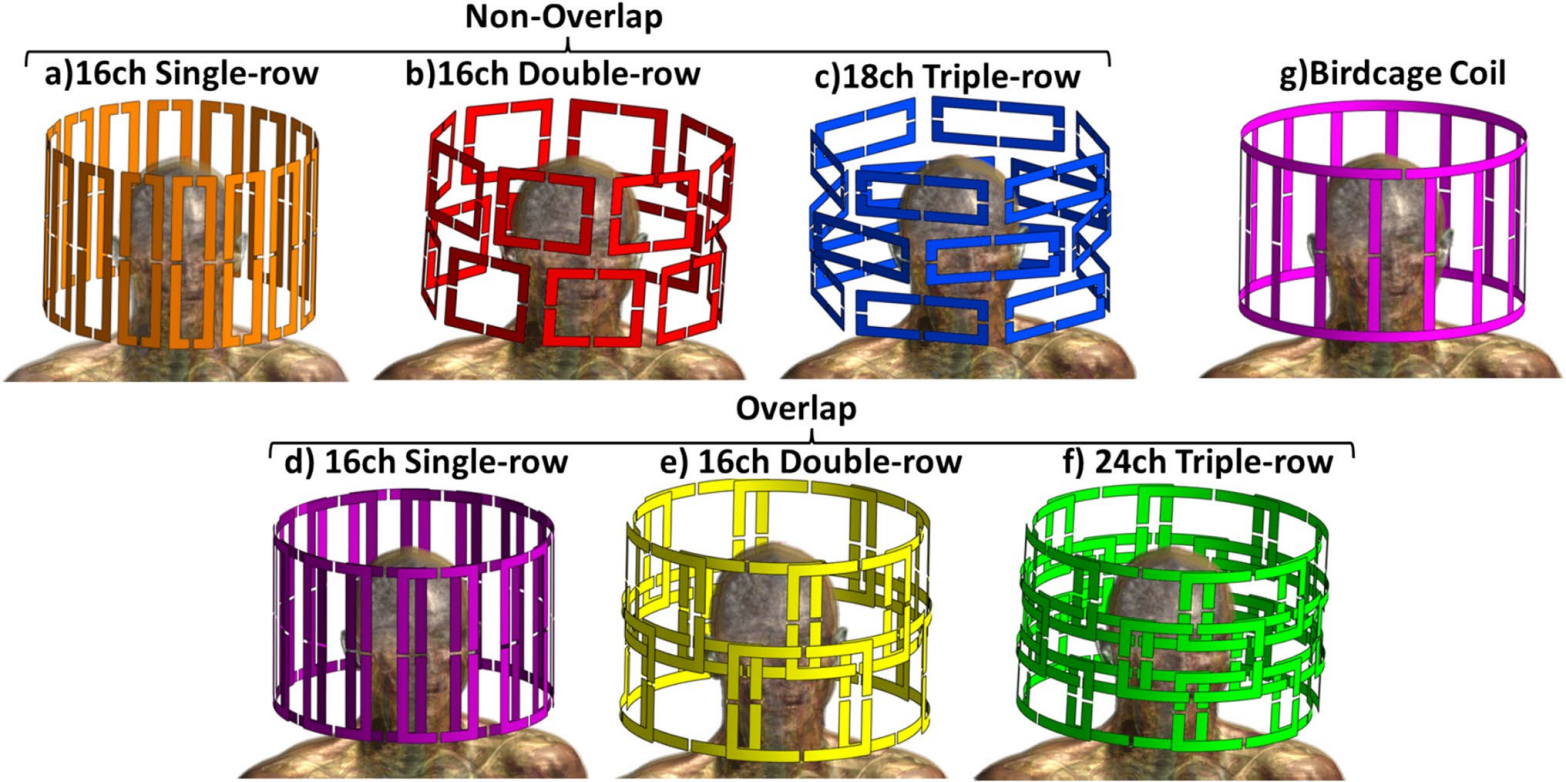
RF coil designs of (**a**) 16-channel (16ch) single-row non-overlapping, (**b**) 16ch double-row non-overlapping, (**c**) 18ch triple-row non-overlapping, (**d**) 16ch single-row overlapping, (**e**) 16ch double-row overlapping (**f**) 24ch triple-row overlapping pTx array and of (**g**) birdcage for human head and their dimensions. All pTx coils and birdcage cover the same height and at similar diameters

The study includes six distinct pTx head coil setups consisting of arrays of rectangular loops on a common cylindrical geometry (360 mm in diameter and 250 mm in height) for operation at 3T modelled using Sim4Life (ZMT, Switzerland). The coil arrays are single-row (16-channel (16ch) (16 × 1), overlapping and non-overlapping), double-row (16ch (8 × 2), overlapping and non-overlapping), and triple-row (18ch (6 × 3), non-overlapping and 24ch (8 × 3), overlapping) (Fig. 1a–f). The non-overlapping designs used planar loops, while overlapped coils required all elements to have a curved cylindrical geometry. A circularly polarised 16-rung low-pass birdcage coil with the same dimensions is used for comparison (Fig. 1g). These coils are accommodated inside a cylindrical radiofrequency shield measuring 1560 mm in length and 752 mm in diameter to simulate the MRI scanner bore. All coil elements were modelled using a flat conductor of width 15 mm, defined as a perfect electric conductor (PEC).

The inner dimensions (width x height) of the loops for each pTx coil setup are as follows: for non-overlapping coils, the dimensions are 60 mm × 250 mm for single row, 114 mm × 124 mm for double rows, and 68.5 mm × 181 mm for triple rows. For overlapping coils, the dimensions are 110 mm × 250 mm for single row, 147 mm × 190 mm for double rows, and 110 mm × 190 mm for triple rows. Each loop has a capacitor situated in a 5 mm gap at the centre of each side. Voltage sources are located at the top (cephalic end) of each loop element for the single-row coil, but for double-row and triple-row coils, the voltage sources are positioned on the left-hand side of each loop; coaxial feed cables (and their traps) are excluded as these are likely to have variable paths in any built coil. The birdcage coil has a capacitor set within a 5 mm gap at the centre of each leg. Additionally, two voltage sources are positioned on the top ring of the birdcage coil, one placed at 0° directly anterior and the other at 90° in the anticlockwise direction relative to the first source as viewed from the cephalic end. The individual loops are arranged equally spaced around circumferential rows. For arrays composed of multiple rows, these are displaced symmetrically along the z-axis, with the elements in each row offset circumferentially by half a loop diameter relative to those in the next row. Loop size and placement are chosen to achieve either non-overlapping geometries or to overlap in both horizontal and vertical directions as needed to maximise decoupling. For overlapping coils, the ratio between the overlapping distance of the adjacent loops was chosen as 12% following the suggested margin as 10–14% [32, 33].

**Fig. 2:**
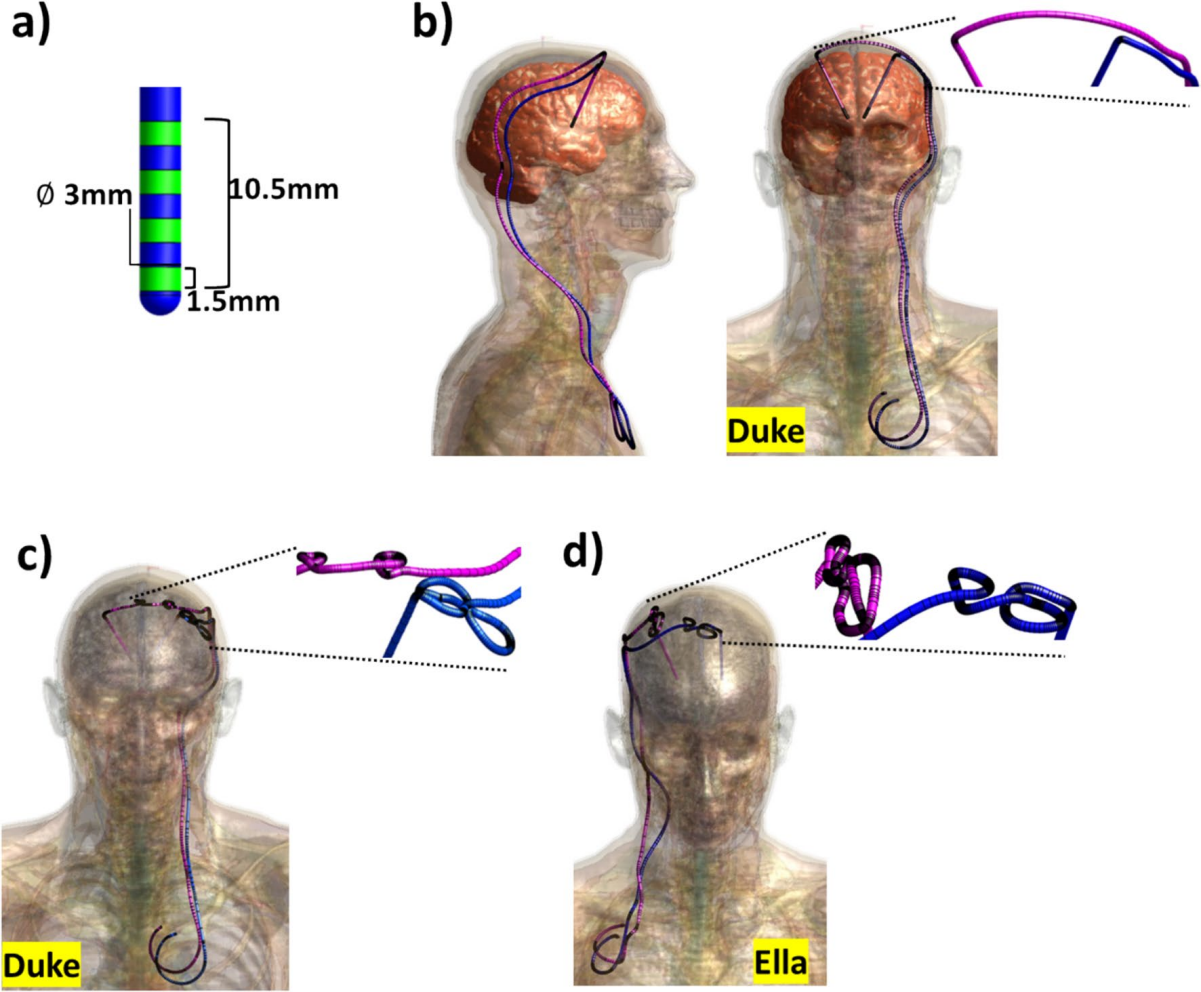
DBS model with (**a**) the 4 electrodes (Medtronic model 3387) placed to the tip of the leads DBS, (**b**) simplified model without extracranial looping on Duke human model, (**c**) extracranial looping added, keeping the lead trajectory same as (**b**) on Duke, (**d**) a new lead trajectory including extracranial looping with the female human model Ella. The designated area of the models represents the region utilised for SAR calculation through the use of Q-matrices. The brain volume, highlighted in orange (**b**), delineates the area where B_1_^+^ optimisation occurs

The realistic human model Duke [34] was used for all simulations. It was placed brain-centred into each coil/array. For simulations with bilateral DBS leads, electrodes and extension wires leading down to the chest were added. The IPG needed to supply the DBS electrodes, which is normally placed in the chest wall a little below the collarbone, so well outside the coils being modelled, was not included in the simulations. The present study focuses on lead and electrodes (Fig. 2). The lead is made up of a wire enclosed in an insulator, which connects the electrodes at the tip. These were modelled as four exposed 1.5 mm radius cylindrical pads, 1.5 mm in length and 1.5 mm apart, approximately simulating DBS 3379 (Medtronic Inc., Minneapolis, USA). We obtained an example trajectory for a unilateral DBS lead targeting the subthalamic nucleus (STN) from Medtronic (private communication). The trajectory starts at the electrode in the target area and terminates just below the collarbone. We created a bilateral DBS model by mirroring the intra-cranial portion of the lead trajectory to place electrodes in the opposite lobe of the brain but following a similar extracranial trajectory for the remainder of the lead. For the sake of computational efficiency, the lead trajectory at the top of the head was simplified to a rather straight trajectory instead of complex sub-cutaneous lead loops that can occur in clinical practice. The conductive wire thickness is 0.5 mm in radius. A difference in electrode design compared to the original model is that the insulator thickness is set to 1 mm, as the actual 0.135 mm thickness was not computationally feasible due to the extremely fine meshing it would require around the DBS leads. The DBS insulator was modelled as a dielectric with a relative permittivity of 4 and no electrical conductivity. DBS electrodes and wire were also modelled as PEC.

Furthermore, two additional scenarios are investigated to observe the effects of diversity in DBS and human models: 1) on the Duke model, featuring the same DBS trajectory with extracranial looping added at the top of the head (Fig. 2c), and on the female human model, Ella, using different trajectories that also include extracranial looping moving down to a pulse generator placed on the opposite side of the body (Fig. 2d).

Non-uniform computational grids were used for all simulations. Initially, all coils were simulated without DBS electrodes using ~ 45M (340 × 230x590) cells with sizes ranging from 1.7 × 1.7 × 1.8 mm^3^ to 83 × 79 × 85 mm^3^. After comparing performance without electrodes, only the overlapping coils were simulated with electrodes present, which required a finer mesh of ~ 180M (500 × 460 × 800) cells with sizes ranging from 0.4 × 0.3 × 0.4 mm^3^ to 108 × 103 × 116 mm^3^. Matched simulations were then done for the overlapping coils without the DBS electrodes present. A semi-automated voxel checker [35] was utilised to ensure the integrity of the models, specifically to prevent DBS conductor wire leakage beyond the borders of the insulator. Absorbing boundary conditions were employed at the simulation space boundaries to prevent interference from reflected waves with the propagating electromagnetic wave.

For each coil, multiport simulations with Gaussian excitation at 123 MHz and 50 Ω impedance were performed, treating ports only (including lumped elements as ports) for tuning, matching, and decoupling purposes. The exported multiport impedance data were then used in a co-simulation method (Optenni Ltd., Finland) to optimize the values of lumped elements (defined as lossless) [36]. The resulting co-simulation circuit values, including lumped elements as series/parallel capacitors, were applied in a second multiport simulation with harmonic excitation at 123 MHz applied only on the loop coil drive ports and 50 Ω impedance to obtain the S-matrix and field data.

### Evaluation of the simulation data

Individual B_1_^+^ and electric fields were extracted on a head and chest sensor volume (201 × 251x401 mm^3^) (Fig. 2a), resampled to a 1 mm isotropic image grid, and exported to Matlab (MatWorks, Inc.).

Q-matrices [37] were computed from simulated electric fields over a more limited region of 201 mm from left to right. The area outside of this region was tested, and it was observed that the change in SAR was negligible due to the position of the coil. One-gram tissue mass-averaging is used to assess the local SAR (SAR_1g avg_). A virtual observation point (VOP) set [38] derived from these Q-matrices was created with an overestimation of 12.5% using compression software provided by Siemens Healthcare (Erlangen, Germany). The number of VOPs varies between 3 and 15 for the coils with DBS, while for those without DBS, this value ranges between 23 and 122. The Q-matrices were downsampled by a factor of 3 in a staircase approach to facilitate this compression step. The fidelity of this procedure was assessed using the double-row coil by checking SAR prediction for 1 million random shims, where the mean deviation between the original and the downsampled data remained less than 0.01%.

The simulated B_1_^+^ distributions and VOPs were used to study the performance of each coil design for the Duke model without and with the DBS. Amplitude/phase shimming was performed for each coil design using magnitude least squares (MLS) optimisation regularised by local SAR [39] to achieve a uniform target field (Eq. 1):

1 $$\underset{{\varvec{x}}}{\mathbf{min}}\{{\left||\left|{\varvec{A}}{\varvec{x}}\right|-{\varvec{b}}\right|}_{{\varvec{\Omega}}}{|}_{2}^{2}+{\varvec{\lambda}}{{\varvec{S}}{\varvec{A}}{\varvec{R}}}_{{\varvec{m}}{\varvec{a}}{\varvec{x}},\boldsymbol1{\varvec{g}},{\varvec{a}}{\varvec{v}}{\varvec{g}}{ }}\}$$

In Eq. 1, the first term is the magnitude of the difference between a uniform target, b, of 1μT and the current predicted B_1_^+^ spatial distribution Ax, obtained by the application of complex weights, x, to the per coil element transmit B_1_^+^ maps, A. The absolute value |Ax| is calculated as the spatial phase variation of the shimmed B_1_^+^, which is unimportant for many MRI applications. A region of interest was applied to limit all field assessments to the brain only (the orange area in the human model shown in Fig. 2b). The second term in Eq. 1 is the maximum SAR value, calculated using VOP. The algorithm constantly picks the highest local SAR value for the current weights x. During the optimisation, the sum of these terms is minimised by varying the shims, x. To encounter the challenges that might be caused by the nature of the non-convex optimisation, the optimisation is executed 100 times.

The balance between the homogeneity term and the SAR term is set by a Tikhonov regularisation parameter. By varying between 0.1 and 90, L-curves demonstrating the trade-off between excitation error (plotted as root magnitude means square error (RMMSE)) and local SAR were obtained. These L-curves were used to select optimal shim conditions for comparison (Fig. 4). Performance was assessed after RF shimming by constructing histograms of B_1_^+^ values over the brain region of interest, examining maximum intensity projections of both B_1_^+^ and local SAR. While the SAR_1g avg_ is used to observe focal heating in simulations with and without DBS [40], the SAR_10g avg_ is observed in simulations without DBS to align with standard practices according to the IEC 60601-2-33:2022 guidelines [12]. The average SAR for the whole head, SAR_Whole-Head-Avg_, defined as the region from just below the chin to the apex of the human model's head, was also calculated for each shimmed test condition.

## Results

**Fig. 3:**
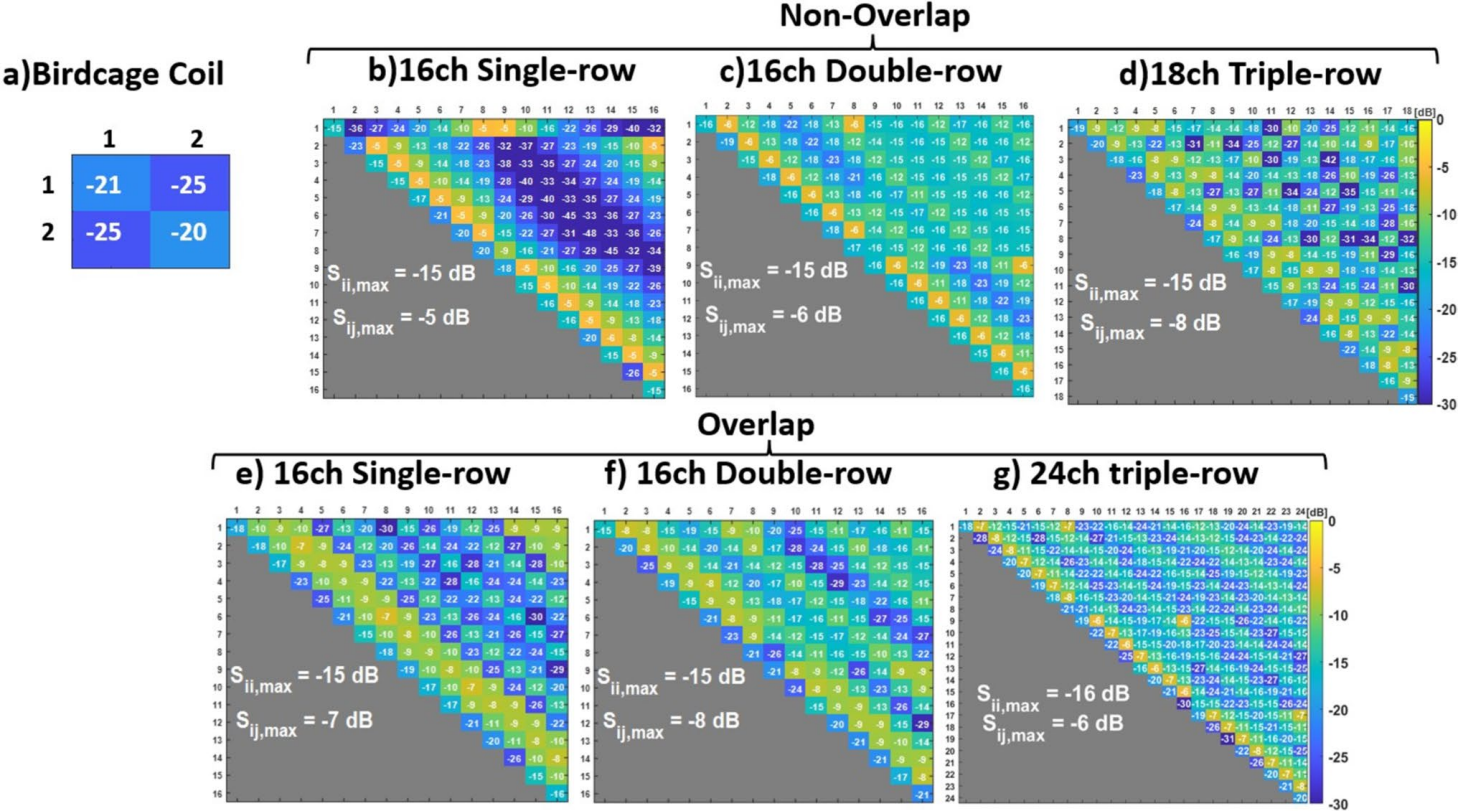
The simulated S-matrices of (**a**) birdcage, (**b**) 16-channel (16ch) single-row non-overlapping, (**c**) 16ch double-row non-overlapping, (**d**) 18ch triple-row non-overlapping, (**e**) 16ch single-row overlapping, (**f**) 16ch double-row overlapping, (**g**) 24ch triple-row overlapping pTx array for the human head

Figure 3 shows the scattering parameters presented as matrices for all port combinations for all coils for the models without the DBS included. The maximum reflection coefficients for the birdcage, non-overlapping, and overlapping coils are − 20 dB, − 15 dB, and − 15 dB, respectively. The birdcage coil displayed a lower coupling coefficient between channels of − 25 dB, in contrast to the pTx coils, which revealed variations between − 8 dB and -5 dB. When the DBS was added to the models, there were only minor variations in scattering parameters (< -1 dB for the maximum reflection and coupling coefficients). All results are presented for the coils tuned and matched without electrodes, as would be the case in practice.

**Fig. 4:**
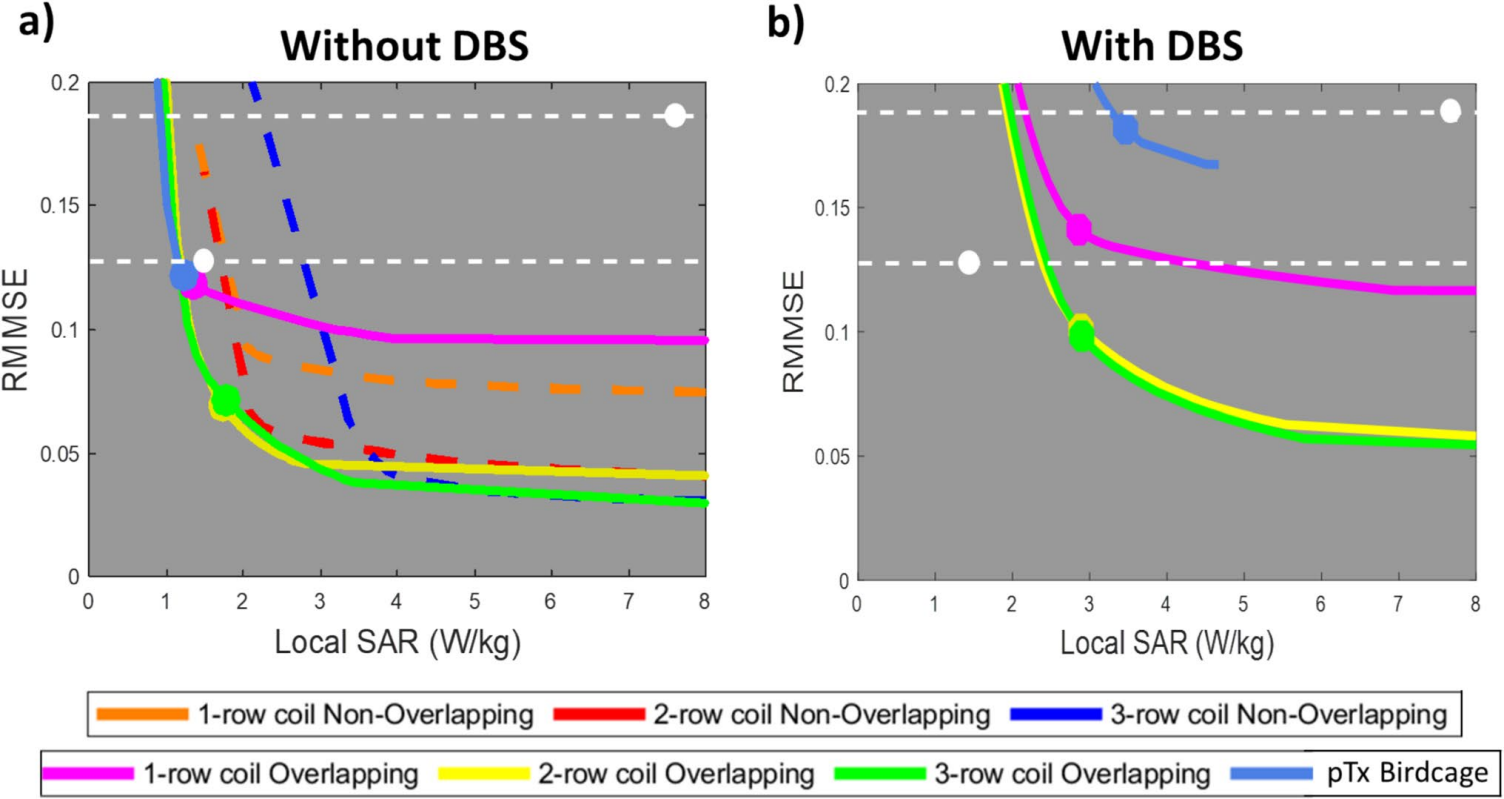
L-curve plot of root magnitude means square error (RMMSE) versus Local SAR (calculated using VOPs) for (**a**) all pTx coils without DBS and for (**b**) the overlapping coils with DBS. Each curve is generated by performing the SAR-regularised RF shimming calculation for multiple values of the regularisation parameter, which varies between 0.5 and 90. Circular markers indicate the optimal corner points or the optimal shims. The white dashed line denotes comparable excitation errors to the birdcage coil without and with DBS. The white circular dots represent the error versus local SAR for the birdcage coil (**a**) without DBS and (**b**) with DBS

Figure 4 shows L-curves that display the trade-off between B_1_^+^ homogeneity error and local SAR (calculated using VOPs) for each coil without (Fig. 4a) and with (Fig. 4b) DBS. Figure 4a shows that for multi-row coils, the overlapping designs (solid lines) outperform the non-overlapping variants (dashed lines). For the single-row coil designs, the L-curves cross, with the overlapping design, achieved the lowest absolute SAR values, but the non-overlapping design achieved better homogeneity (lower RMMSE). Given the general trend for overlapping coils to perform better when no electrodes are present, these designs were selected for simulation with DBS in situ. Figure 4b shows that the performance of the double- and triple-overlapping coils were virtually identical, and both offered a superior balance between homogeneity and local SAR than the single-row coil. As a point of reference, the quadrature birdcage coil achieved a RMMSE of 0.125 μT without DBS in place and 0.192 μT with DBS in situ, combined with local SAR values of 1.5 W kg^−1^ μT^−2^ and 7.3 W kg^−1^ μT^−2^, respectively (white dots in Fig. 4). The birdcage performance without and with DBS is indicated by dashed lines in both Fig. 4a, b. All the array coils could achieve improved homogeneity compared to the quadrature and pTx birdcage coil with DBS, which could be achieved at lower SAR except in the limit of low local SAR (extreme left of the graphs in Fig. 4).

**Fig. 5:**
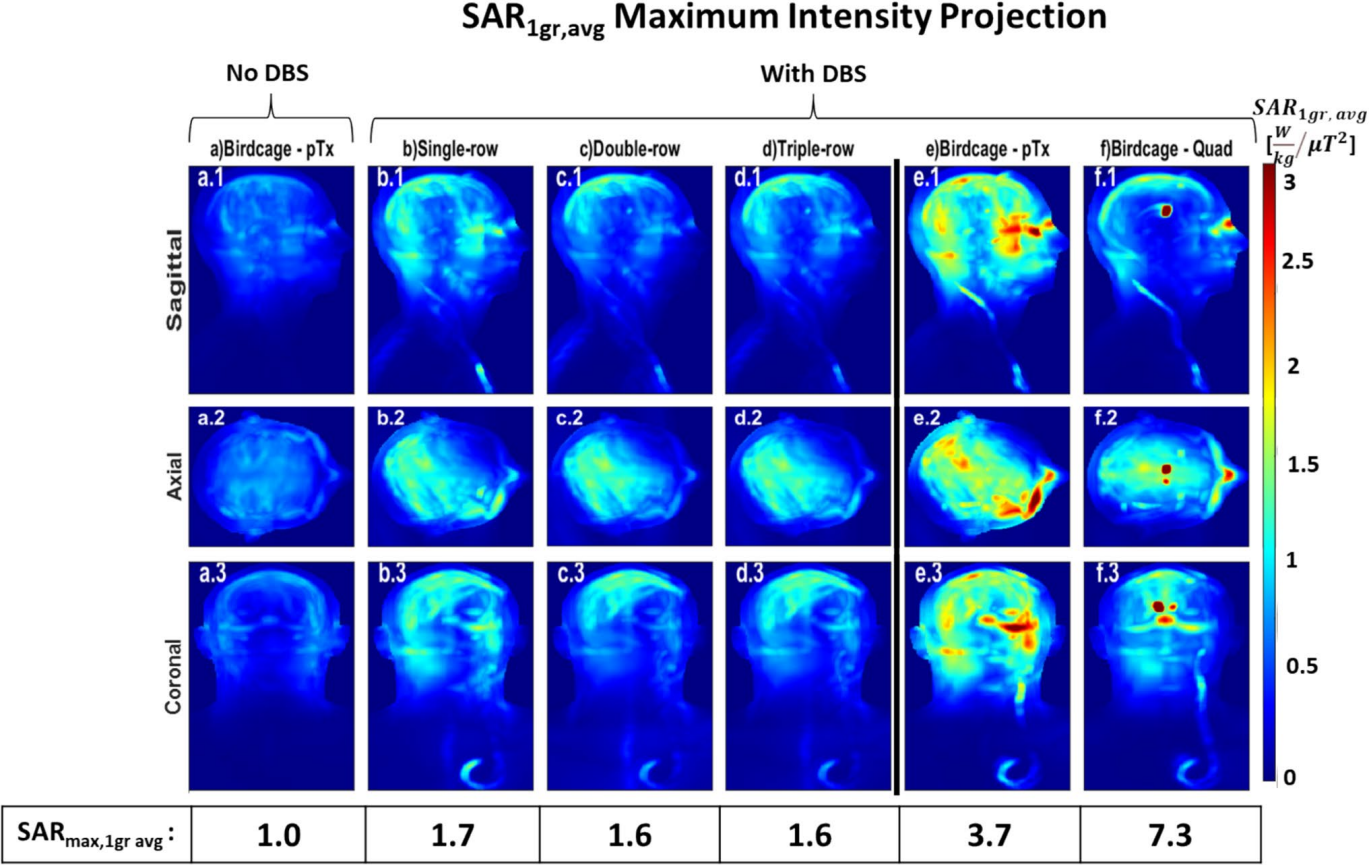
SAR_1g avg_ (W/kg/μT^2^) maximum intensity projection (MIP) maps when the RF excitation errors are equal to the birdcage: without DBS for (**a**) birdcage coil in pTx mode; with DBS for (**b**) 16ch single-row, (**c**) 16ch double-row, (**d**) 24ch triple-row overlapping coils and birdcage coil in (**e**) pTx mode and (**f**) quadrature mode normalised to B_1_^+^_mean_ over the whole brain

The following performance comparisons can readily be achieved, all referenced to creating a 1 μT mean B_1_^+^ field over the whole brain:

Considering the absence of DBS, upon adjusting shims for the multi-row PTx coils to match the B_1_^+^ homogeneity of the quadrature birdcage coil with and without DBS in situ (RMMSE = 0.192 μT and 0.125 μT, respectively), typical of a conventional MRI scanner for conventional imaging examinations (Fig. 5a), all pTx coils with overlapping elements and the pTx birdcage coil exhibited a similar SAR_max,1g avg_ of 1 W kg^−1^ μT^−2^. With the optimised points (optimal shims) selected from each L-curve for each coil (Fig. 7a-d), the multi-row coils produced superior homogeneity at 42% lower COV than the single-row and pTx birdcage coils and 46% lower than quadrature birdcage coil. The pTx coils and pTx birdcage coil provided the lowest SAR_max,1g avg_ (1 W kg^−1^ μT^−2^), 33% lower than the quadrature birdcage coil (Fig. 8). In the absence of DBS, the SAR_Whole-Head-Avg_ of all overlapping pTx coils exhibits values comparable to that of the birdcage coil. The single-row, double-row, and triple-row configurations demonstrated SAR_Whole-Head-Avg_ of 0.27, 0.23, and 0.22 W kg^−1^ μT^−2^, respectively, while the birdcage coil presented a value of 0.26 W kg^−1^ μT^−2^. The SAR_max, 10g avg_ for the single-row, double-row, triple-row coils, and birdcage coil in pTx and quadrature mode are 0.7 W/kg, 0.5 W/kg, 1.2 W/kg, 0.8 W/kg and 1.1 W/kg, respectively.

**Fig. 6:**
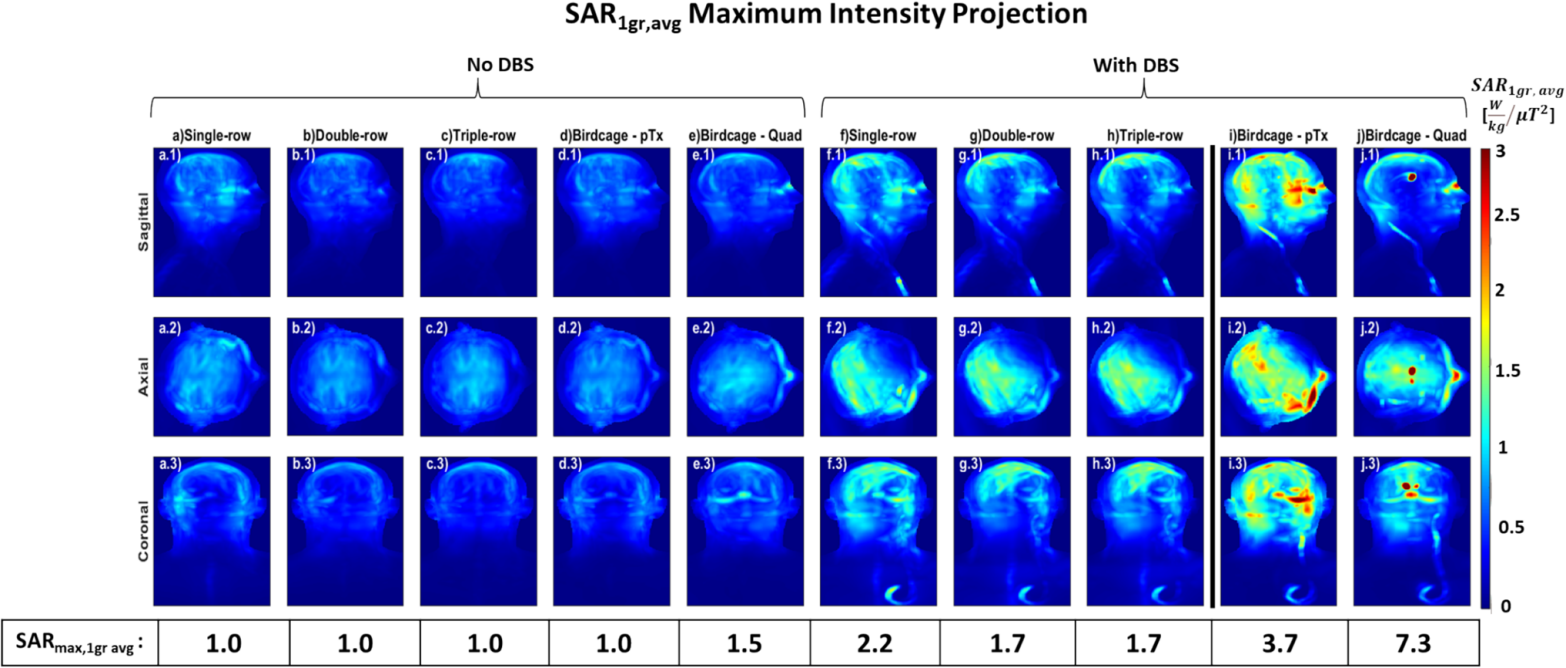
SAR_1g avg_ (W/kg/μT^2^) maps using shims optimised to achieve the same the RF excitation errors as the birdcage with DBS for all coils: without DBS for (**a**) 16ch single-row, (**b**) 16ch double-row, (**c**) 24ch triple-row overlapping coils, birdcage coil in (**d**) pTx mode and (**e**) quadrature mode; with DBS for (**f**) 16ch single-row, (**g**) 16ch double-row, (**h**) 24ch triple-row overlapping coils and, birdcage coil in (**d**) pTx mode and (**e**) quadrature mode, normalised to B_1_^+^_mean_ over the whole brain

**Fig. 7:**
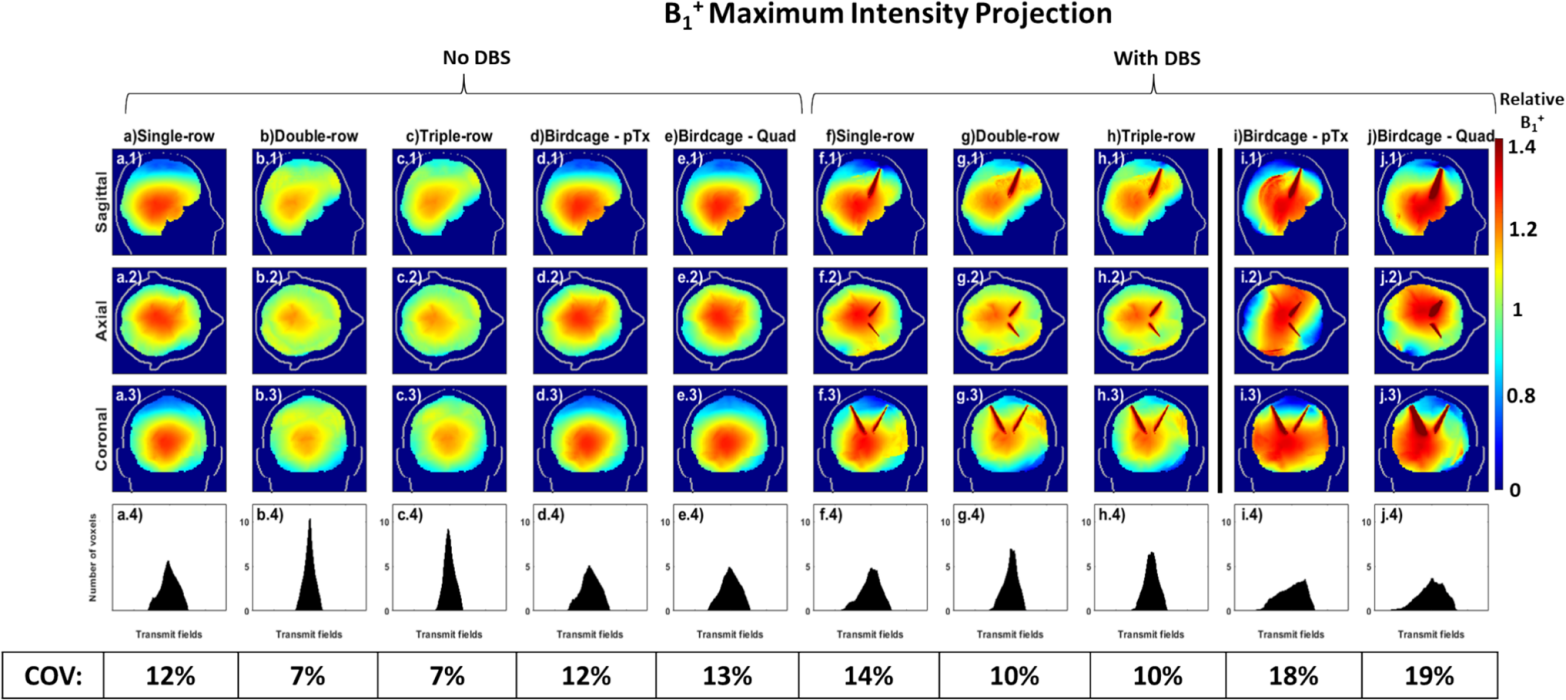
B_1_^+^ MIP maps using optimised shims selected at the ‘corner’ of the L-curves in Fig. 3: without DBS for (**a**) 16ch single-row, (**b**) 16ch double-row, (**c**) 24ch triple-row overlapping coils, birdcage coil in (**d**) pTx mode and (**e**) quadrature mode; with DBS for (**f**) 16ch single-row, (**g**) 16ch double-row, (**h**) 24ch triple-row overlapping coils and birdcage coil in (**i**) pTx mode and (**j**) quadrature mode, (**a**–**j**. 4) histograms of distribution of transmit fields, coefficient of variation values (s(|B_1_^+^|)/μ(|B_1_^+^|)), normalised to B_1_^+^_mean_ over the whole brain

**Fig. 8:**
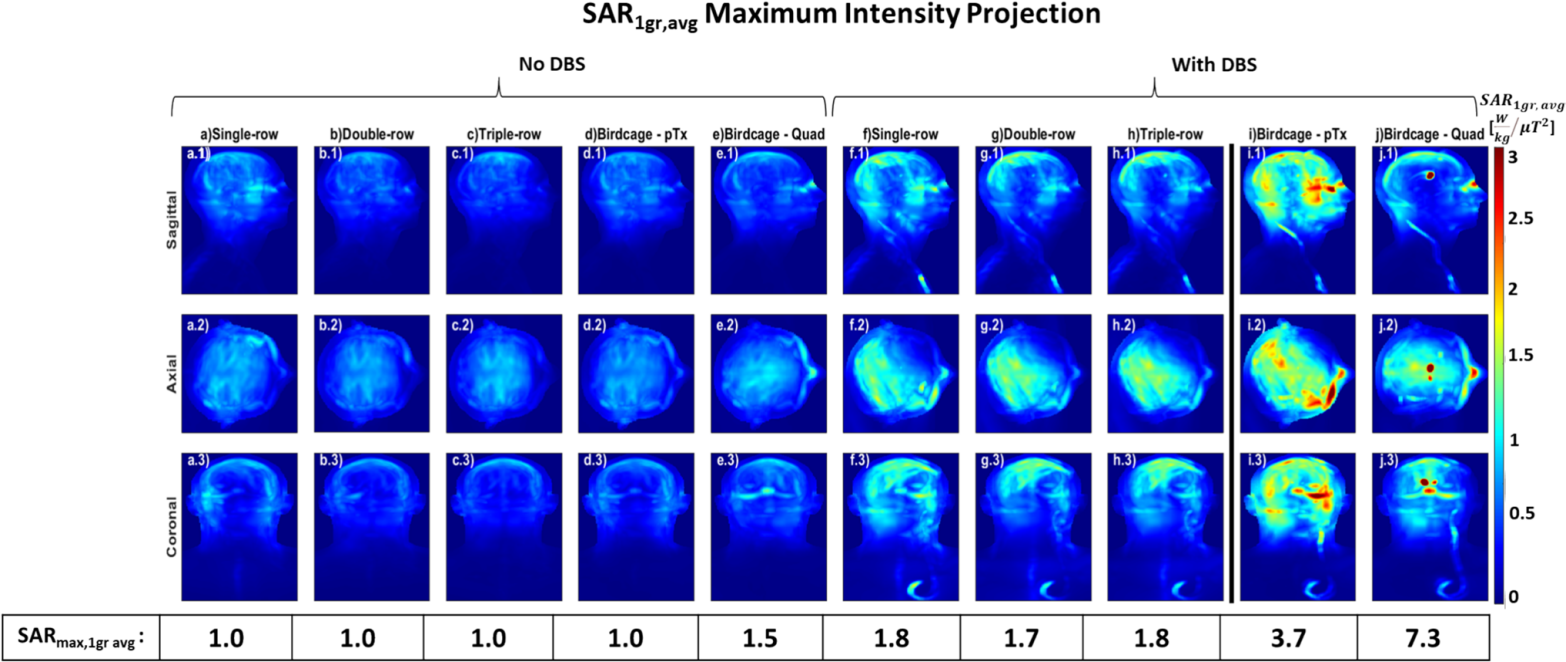
SAR_1g avg_ (W/kg/${{\mu T}}^{2}$) MIP maps using optimised shims selected at the ‘corner’ of the L-curves: without DBS for (**a**) 16ch single-row, (**b**) 16ch double-row, (**c**) 24ch triple-row overlapping coils, birdcage coil in (**d**) pTx mode and (**e**) quadrature mode with DBS for (**f**) 16ch single-row, (**g**) 16ch double-row, (**h**) 24ch triple-row overlapping coils and birdcage coil in (**i**) pTx mode and (**j**) quadrature modem, normalised to B_1_^+^_mean_ over the whole brain

Considering the presence of DBS, upon adjusting shims to match the B_1_^+^ homogeneity of the birdcage with DBS, both double-row and triple-row multi-row coils exhibited a SAR_max,1g avg_ of 1.6 W kg^−1^ μT^2^ (Fig. 5c–d). This represented a reduction of 6% compared to the single-row coil and was 57% and 78% lower than the pTx and quadrature birdcage coil with DBS (7.3 W kg^−1^ μT^−2^), respectively and, in fact, was only 7% higher than the quadrature birdcage coil without DBS (1.5 W kg^−1^ μT^−2^). The single-row coil showed a SAR reduction of approximately 54% and 77% compared to the pTx and quadrature birdcage coil, respectively. Matching the B_1_^+^ homogeneity of the birdcage coil without DBS, the double-row and triple-row coils had a SAR_max,1g avg_ of 1.7 W kg^−1^ μT^2^ marking 54% and 77% lower values than the pTx and quadrature birdcage coil with DBS, respectively (Fig. 6f–j). The multi-row coils produced SAR value 23% lower than the single-row coil, which itself achieved 41% and 70% lower values than the pTx and quadrature birdcage coils with DBS, respectively. With the optimised shims selected from each L-curve, the multi-row coils achieved a COV of 10%, reflecting a 29% improvement in homogeneous excitation compared to the single-row coil and a ~ 46% improvement over the pTx and quadrature birdcage coils with DBS (Fig. 7i, j). Figure 8 reveals that the overlapping pTx coils manifested a SAR_max,1g avg_ of 1.7–1.8 W kg^−1^ μT^−2^, which is a ~ 53% and ~ 76% reduction compared to the pTx and quadrature birdcage coil with DBS, respectively. With DBS in place, the SAR_Whole-Head-Avg_ for overlapping pTx coils is close to that of the birdcage coil. The single-row had a SAR_Whole-Head-Avg_ of 0.33 W kg^−1^ μT^−2^, the double-row 0.27 W kg^−1^ μT^−2^, the triple-row 0.28 W kg^−1^ μT^−2^, compared to the birdcage coil’s 0.32 W kg^−1^ μT^−2^.

**Fig. 9:**
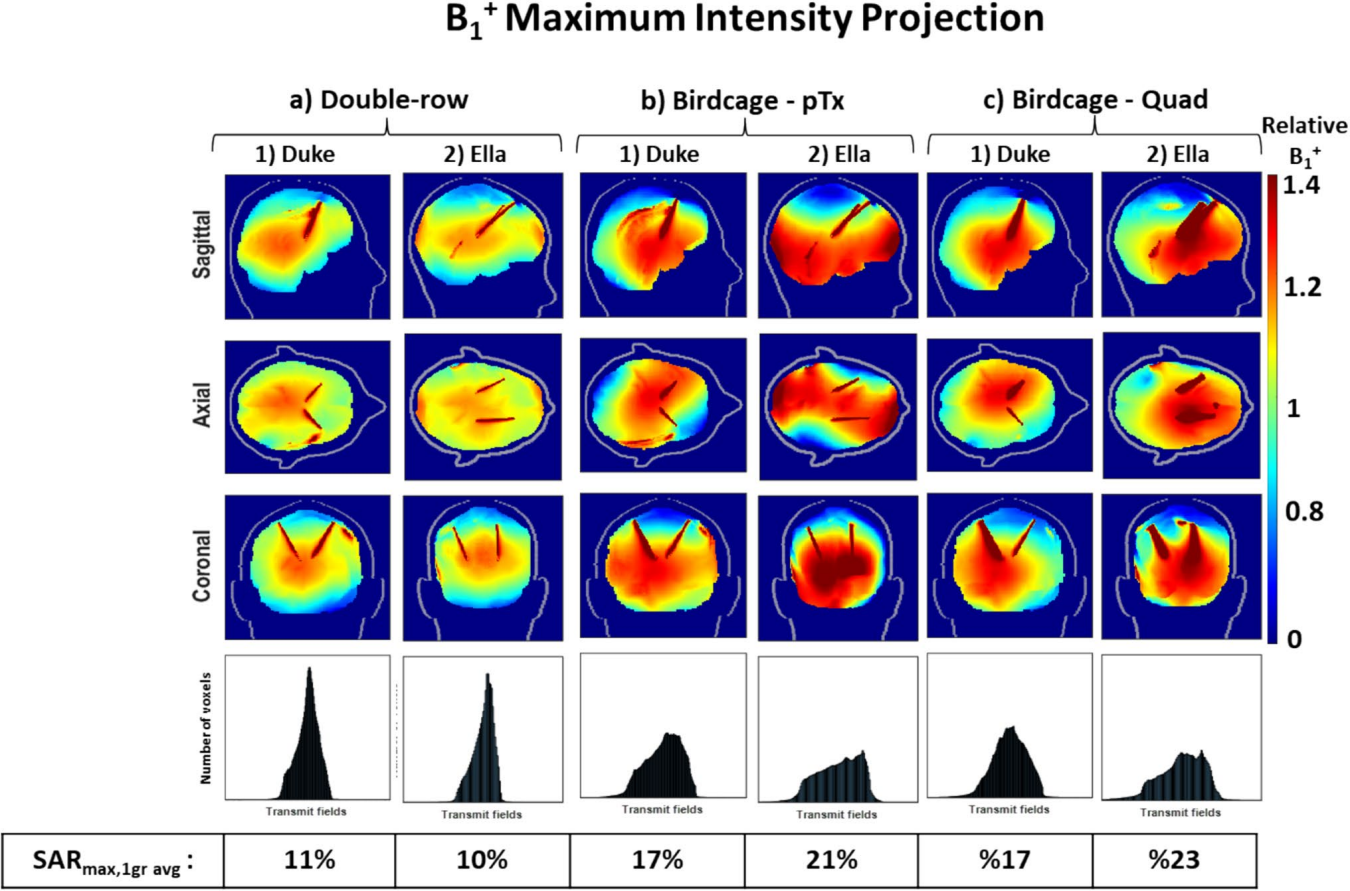
B_1_^+^ MIP maps with shims representing the 'corner' of the L-curves using DBS models with extracranial looping for (**a**) 16ch double-overlapping coil on (1) Duke and (2) Ella, for (**b**) birdcage in pTx (dual-drive) mode on (1) Duke and (2) Ella and for (**c**) birdcage coil in quadrature mode on (1) Duke and (2) Ella, histograms of distribution of transmit fields given in the 4th row, coefficient of variation values (s(|B_1_^+^|)/μ(|B_1_^+^|)), normalised to B_1_^+^_mean_ over the whole brain

When comparing different DBS trajectories with extracranial looping (Fig. 2c-d) placed in a double-row coil and configured with optimised shims, the coil achieved a B_1_^+^ field over the whole brain with COV of 10–11% for both models (Fig. 9a). These findings are consistent with the COV of 10% (Fig. 7g) for the double-row coil on a DBS model without extracranial looping (Fig. 2b). While double-row coil with extracranial looping on Duke (Fig. 9.a.1) exhibits a 35% improvement in homogeneous excitation compared to the pTx and quadrature birdcage coil (Fig. 9.b.1 and Fig. 9.c.1), double-row coil with extracranial looping on Ella (Fig. 9.a.2) decreases the COV by ~ 54% compared to the pTx and quadrature birdcage coil (Fig. 9.b.2 and Fig. 9.c.2).

**Fig. 10:**
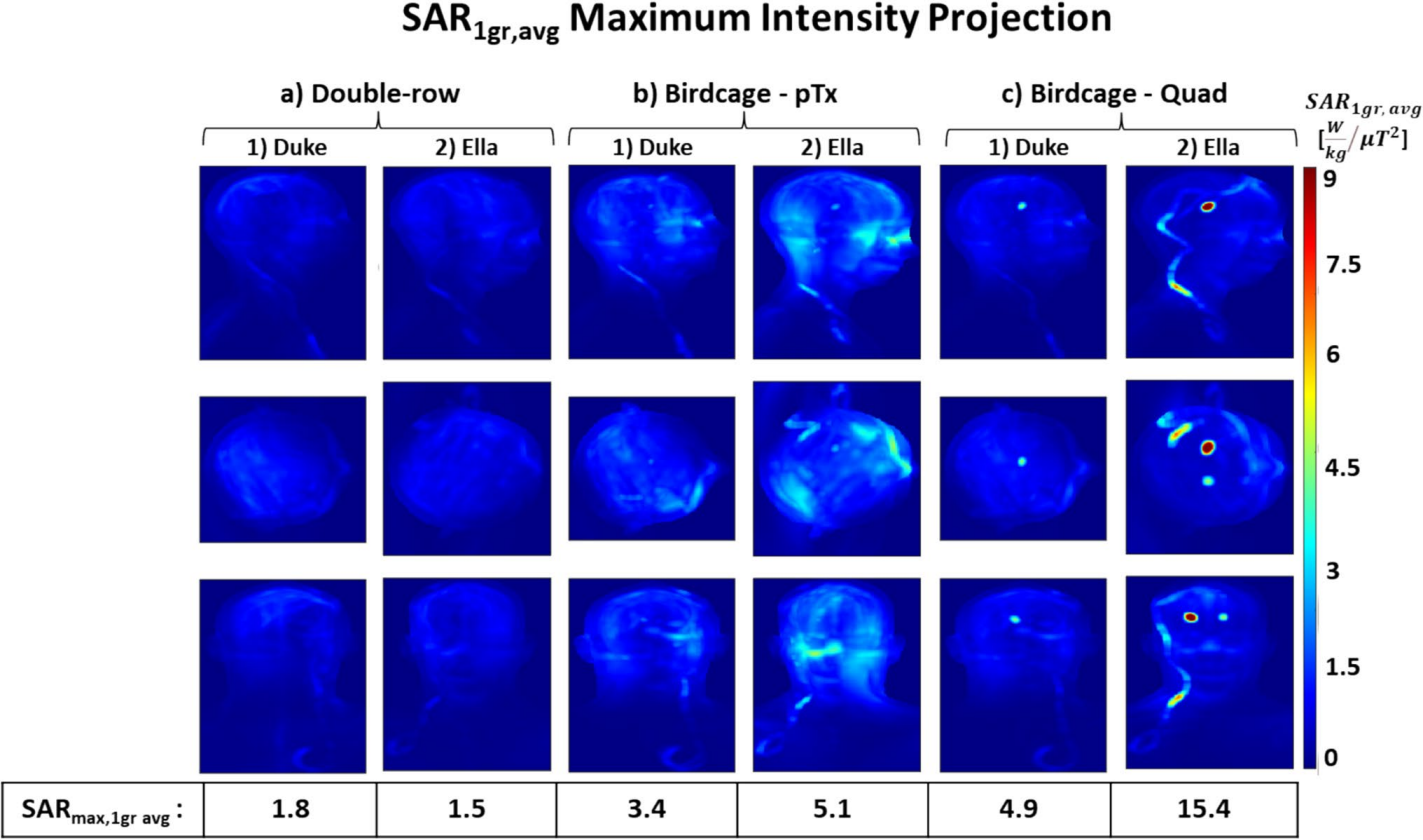
SAR_1g avg_ (W/kg/${{\mu T}}^{2}$) MIP maps with shims representing the ‘corner’ of the L-curves using DBS models with extracranial looping for (**a**) 16ch double-overlapping coil on (1) Duke and (2) Ella, for (**b**) birdcage in pTx (dual-drive) mode on (1) Duke and (2) Ella and for (**c**) birdcage coil in quadrature mode on (1) Duke and (2) Ella, normalised to B_1_^+^_mean_ over the whole brain

Figure 10 shows that the double-row coil with extracranial looping on Duke resulted in a SAR_max,1g avg_ of 1.8 W kg^−1^ μT^−2^, similar to the case without extracranial looping. The double-row coil with extracranial looping on Ella exhibited a SAR_max,1g avg_ of 1.5 W kg^−1^ μT^−2^, marking approximately a 16% reduction compared to the double-row coil with Duke for both simple and more complex extracranial wire geometry. While the double-row coil with extracranial looping on Duke showed a SAR reduction of approximately 47% and 63% compared to the pTx and quadrature birdcage coil, respectively, the double-row coil with extracranial looping on Ella reduced the local SAR approximately 71% and 90% compared to the pTx and quadrature birdcage coil, respectively.

## Discussion

The aim of this simulation study was to evaluate the performance of parallel-transmit head transmit RF coils compared with a circularly polarised (quadrature) and dual-drive (pTx) head birdcage coil on a computational human model without and with implanted bilateral DBS, with a focus on excitation fidelity under SAR control for standard brain imaging targets. Our computations compellingly confirm that multi-row pTx coils: (1) can produce more homogeneous transmit fields than the birdcage coil and can operate at lower local SAR when there are no DBS; (2) can maintain significant benefit by both measures in a straight comparison with DBS; and (3) can achieve local SAR levels in the presence of electrodes that are similar to a quadrature birdcage without any implanted electrodes without sacrificing homogeneity.

RF shimming was performed to assess the coils, optimising for B_1_^+^ homogeneity over the whole brain (rather than over a single slice as is sometimes done) with a peak local SAR penalty term. L-curves, in which the relative weighting of the SAR term was varied (Fig. 4), demonstrated the trade-off between excitation error for B_1_^+^ homogeneity and local SAR for each coil design. In the absence of DBS electrodes, overlapping coil elements, in general, provided a better strategy than using non-overlapping elements, with an exception seen in the single-row pTx coil where the L-curves crossed. In the simulations, while the non-overlapping coil designs utilised planar loops, the overlapping coils were forced to employ a curved cylindrical geometry for all elements. Since the observed performance of the non-overlapping designs was inferior to their corresponding overlapping designs, the non-overlapping designs were not re-modelled using the fully curved geometry and were not modelled with DBS added. Furthermore, it has been verified that the positioning of the ports on the birdcage coil, whether in their current position or in the conventional position offset symmetrically at the back of the head, does not affect the SAR_max,1g avg_ or the COV.

When comparing the L-curves of the coils without DBS, all pTx coils outperformed the pTx and the quadrature birdcage coil. The pTx coils demonstrated improved homogeneity and lower SAR values, enhancing safety and efficiency. Upon introducing the DBS devices, the L-curves for all tested pTx coils shifted upwards to the right, indicating increased inhomogeneity, and heightened local SAR values, although all still achieved more favourable performance than the birdcage. Single-row coil performance was in agreement with the findings by McElcheran et al. [25], who compared an 8-channel single-row coil with the birdcage coil. It was also observed that using the birdcage coil in pTx mode significantly improves the balance between B_1_^+^ homogeneity and local SAR compared to the birdcage coil in quadrature mode. In the no-DBS scenario, the pTx birdcage coil performs similarly to the pTx coils in the lower local SAR and higher COV regime, but when the DBS is in situ, the pTx coils significantly outperformed the pTx birdcage coil.

A striking result is that the multi-row pTx coils could produce more homogeneous fields at slightly higher peak SAR with DBS than is achieved with the birdcage without DBS. Although peak local SAR was markedly reduced, the head average SAR was similar for all coils for all conditions tested (~ 0.5 W/kg lower than the birdcage for multi-row coils and ~ 0.1 W/kg higher for the single-row pTx coil). The capability to lower local SAR even when DBS electrodes are present could be transformative for the brain imaging of this patient group. As an aside, we note that after shimming, the local SAR distributions of pTx designs presented here show peak local SAR values elsewhere in the head and not at the tip of the electrodes (Figs. 5,6,8 and 10), which indicates the importance of a whole head volume SAR assessment.

The triple-row array might have been expected to provide superior RF performance due to its larger number of channels but only showed a minor efficiency benefit compared to the double-row array. Its increased complexity may contribute to higher manufacturing costs and implementation challenges.

A previous study of pTx coils with DBS [26] explored whether adding complexity to the modelled electrodes alters the predicted SAR for a double-row coil across various human and DBS models, and did not find this to be an important aspect. To check stability of results for both our deployed DBS and human model, the double-row coil, which provides optimal performance comparable to the triple-row in this study, is simulated in two additional scenarios: (1) on the Duke model, with added extracranial looping, and (2) on the Ella model, using a different trajectory, also with extracranial looping. It is demonstrated that for the double-row coil, the addition of extracranial looping does not affect B_1_^+^ homogeneity and SAR_max,1g avg_ values for the Duke model (Fig. 9), while for the Ella model, comparable B_1_^+^ homogeneity is achieved, and SAR_max,1g avg_ is reduced (Fig. 10). This helps to reinforce Guerin's findings [26], confirming that the performance of multi-row coils is consistent across different models and unaffected by the specifics of electrode placement or model complexity. In contrast, when we tested this additional trajectory scenario using the birdcage coil, the SAR_max,1g avg_ varied significantly, up to 68%, from one trajectory to another, which aligns with the literature [41]. This unpredictability of the birdcage coil can pose a safety issue, which is mitigated by multi-row pTx coils.

In agreement with the findings presented by McElchearan et al. [23–25], Eryaman et al. [22], and Guerin et al. [26], our study demonstrates that single-row pTx coils outperformed the birdcage coil in the tested configurations. Our research is consistent with the study by Guerin et al. [26], which showed the benefit of two-row pTx head coils over single-row pTx and birdcage coils. In their findings, the heating around the electrodes was reduced by a factor of over 100 when using the double-row head coil while keeping the same level of transmit field homogeneity at the selected slice. A complementary aspect is that they emphasised simulating a broader range of body models [26], while our study focussed on simulating a greater variety of head coil designs.

The pTx coil designs considered are complex and it is challenging to achieve decoupling between individual coils with multiple close neighbours. The achieved coupling coefficients, between -6 dB and -8 dB, could raise a concern in this regard. However, the strong localisation and diversity observed in the transmit fields of the individual channels (Online Resource 1) confirms that coil coupling has not undermined performance. Furthermore, the placement of the voltage sources to the top and left-hand side of the coil elements only resulted in changes in S_ii, max_ and S_ij,max_ parameters of less than 1dB. Another potential risk is that errors in the measured in the coil B_1_^+^ maps or prescribed shims might lead to uncontrolled deviations in performance. To explore this, we performed a test of the sensitivity of the double-row coil with DBS electrodes in situ to phase and amplitude errors in shim measurement or setting. We performed 100 trials, randomly perturbing the optimised shim values by ± 5% in amplitude and phase independently on all channels. This resulted in a maximal increase in SAR_max,1g avg_ of 0.0625% and a maximal reduction of B_1_^+^ homogeneity of 0.3%, supporting a conclusion that performance is robust at least to this level of error.

The simulations performed in this study had some specific limitations. In Figs. 5,6, 8 and 10, with DBS electrodes in place, the optimal shim solutions often have secondary local peaks in SAR at the distal end of the wires in the chest wall, even though the local fields in this location are much lower (~ 70 times) than in the head. We hypothesised that this is an artefact associated with a lack of explicit inclusion of the IPG to provide a termination for the distal end of the DBS lead. To examine this further, one additional simulation for the double-row coil was executed, incorporating an IPG with a conductive case to the inner surface of which the DBS lead's inner conductor is terminated via a 2 kΩ resistor. It was observed that this refinement resulted in only slight changes to field distributions, with a 6% increase in the COV of B_1_^+^ and maximum local SAR, combined with complete eradication of a local peak of SAR in the chest. This provides confidence that the use of more refined models that include the IPG would not change the conclusions of this study. While this study shows substantial potential benefits of pTx for controlling the safety of MRI with implanted electrodes, there remain many challenges in realising these benefits in operational practice. Eryaman and others [19–23] have started to address this challenge, but much is yet to be done. Our results add further motivation.

In conclusion, PTx coils with multiple rows can achieve more homogeneous B_1_^+^ and lower SAR around DBS electrodes at 3T compared to birdcage coils. Together these can achieve SAR efficiency (mean B_1_^+^/$\surd$ *SAR*) that could rival performance in the presence of DBS electrodes of existing coils in the absence of such implants (conventional imaging regime). Single-row pTx coils provide much reduced performance gains. These findings indicate that multi-row pTx coils could offer significant benefits for MRI subjects with DBS electrodes. This provides further motivation for exploring the key challenge of achieving robust deployment of this exciting technology so that its demonstrable potential gains can be delivered in practice for patient benefit.

## Supplementary Information

Below is the link to the electronic supplementary material.

Supplementary file1 (DOCX 202 KB)

Supplementary file2 (STEP 256 KB)

Supplementary file3 (STEP 256 KB)

Supplementary file4 (STEP 253 KB)

Supplementary file5 (STEP 439 KB)

Supplementary file6 (STEP 297 KB)

Supplementary file7 (STEP 548 KB)

Supplementary file8 (PDF 377 KB)

Supplementary file9 (MAT 36 KB)

Supplementary file10 (MAT 134210 KB)

## Data Availability

The available data include a 3D B_1_^+^ map, Virtual Observation Points for the double-row overlapping coil with DBS, the CAD models of all coils and their circuit information which are in the supplementary materials. The SAR-regularised MLS algorithm Matlab code is openly available at https://github.com/RflabLondon/MLSOptimisationCodeSAR
