# Supplementary material for: Design of multi-row parallel-transmit coil arrays for enhanced SAR efficiency with deep brain electrodes at 3T: an electromagnetic simulation study
Source: MAGMA. 2024 Nov 14;38(1):107–20. doi: 10.1007/s10334-024-01212-4 (PMC11790791; doi:10.1007/s10334-024-01212-4)
Supplement: Supplementary file 1 — Supplementary file1 (DOCX 202 KB) [file 10334_2024_1212_MOESM1_ESM.docx]

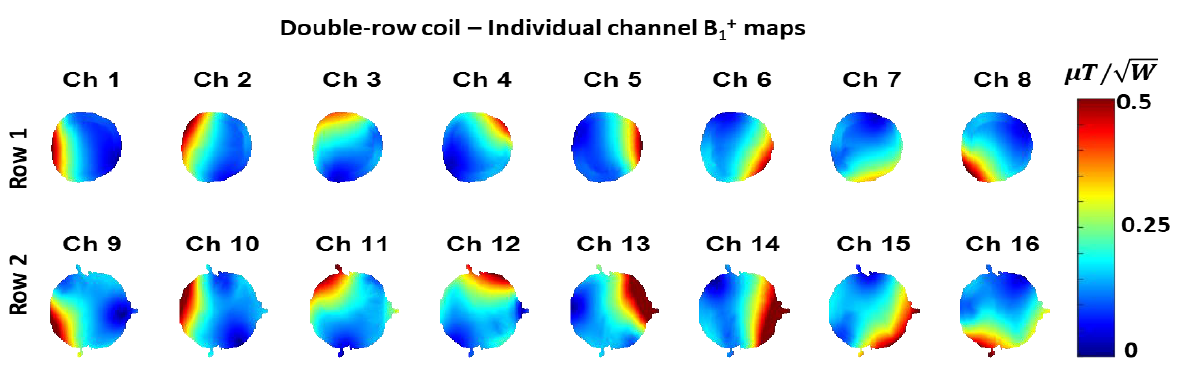


**Online Resource 1** Individual B_1_^+^ field maps at the centre of each row of the 16-channel double-row coil, normalised to 1W input power.
