# Supplementary material for: Design of multi-row parallel-transmit coil arrays for enhanced SAR efficiency with deep brain electrodes at 3T: an electromagnetic simulation study
Source: MAGMA. 2024 Nov 14;38(1):107–20. doi: 10.1007/s10334-024-01212-4 (PMC11790791; doi:10.1007/s10334-024-01212-4)
Supplement: Supplementary file 8 — Supplementary file8 (PDF 377 KB) [file 10334_2024_1212_MOESM8_ESM.pdf]

Single-row Non-Overlapping Coil

Channel Numbering:

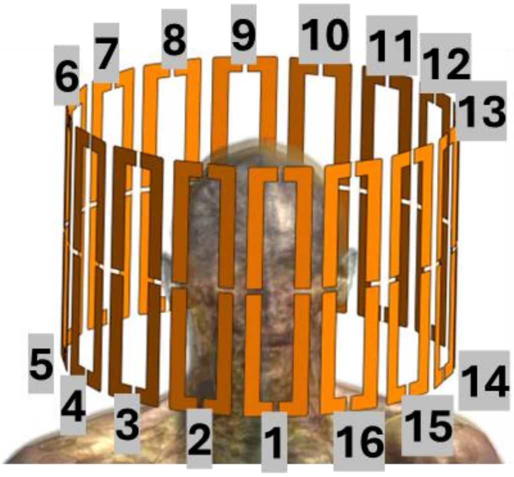

Individual channel circuit diagram:

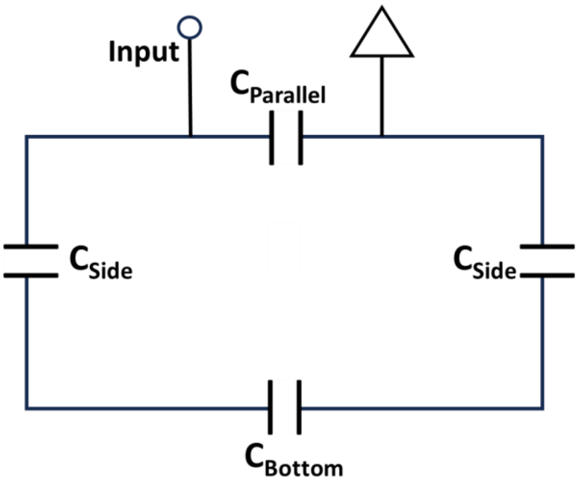

Capacitor values:

Coil Name	$C_{Parallel}$(pF)	C_{Sides}(pF)	C_{Bottom}(pF)
**Loop 1**	46.4	20.5	21.7
**Loop 2**	43.8	25	16.3
**Loop 3**	47.6	23	16.6
**Loop 4**	42.5	29	12.4
**Loop 5**	47.5	15.3	48.2
**Loop 6**	44.5	24.7	14.8
**Loop 7**	44.4	22.5	17.9
**Loop 8**	49.6	18.3	32.3
**Loop 9**	44	18.6	27.5
**Loop 10**	45.1	21.3	21.5
**Loop 11**	45.6	22.6	17.8
**Loop 12**	45.9	26.1	13.9
**Loop 13**	46.7	27.8	12.4
**Loop 14**	43.4	30.8	11.7
**Loop 15**	47.1	18.3	26.7
**Loop 16**	47.1	21.7	20.6

Double-row Non-Overlapping Coil

Channel Numbering:

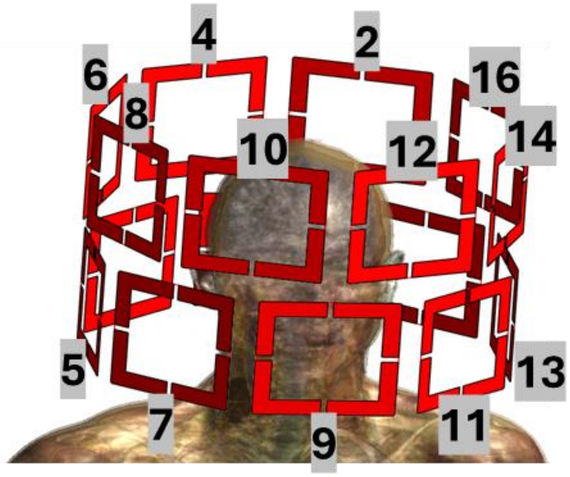

Individual channel circuit diagram:

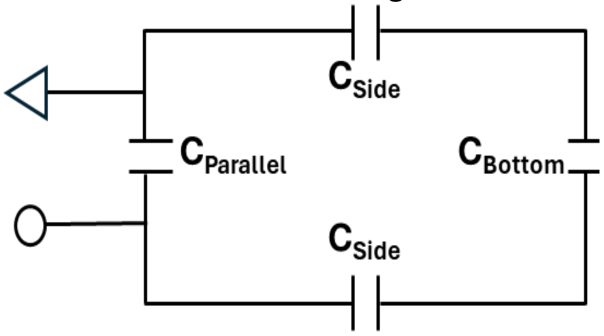

Capacitor values:

Coil Name	$C_{parallel}$(pF)	C_{Sides}(pF)	C_{Bottom}(pF)
**Loop 1 - R1**	80.7	20.7	32.7
**Loop 1 – R2**	78.7	27	18.7
**Loop 2 - R1**	74.3	17.1	71.3
**Loop 2 – R2**	79.9	32.4	15
**Loop 3 - R1**	84.5	22.5	23.5
**Loop 3 – R2**	73.3	17	65.6
**Loop 4 - R1**	77.4	42.6	11.4
**Loop 4 – R2**	81.4	19.5	34.4
**Loop 5 - R1**	79	20.9	35.5
**Loop 5 – R2**	75.2	28.6	17.2
**Loop 6 - R1**	82.2	57.1	9.79
**Loop 6 – R2**	76	18.2	54.1
**Loop 7 - R1**	79.6	39.6	12
**Loop 7 – R2**	80.1	21.1	26.7
**Loop 8 - R1**	76.7	17.4	62.9
**Loop 8 – R2**	77.1	68.2	9.22

Double-row Non-Overlapping Coil

Channel Numbering:

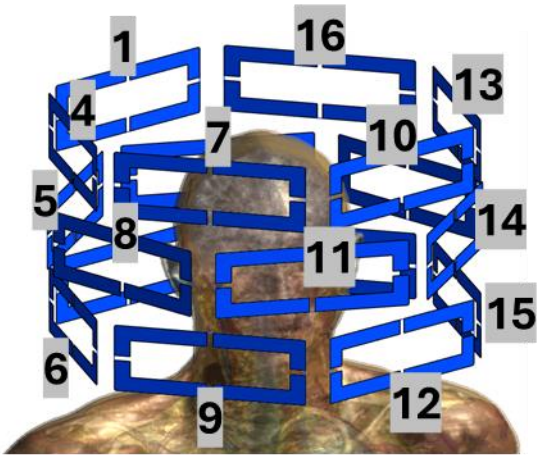

Individual channel circuit diagram:

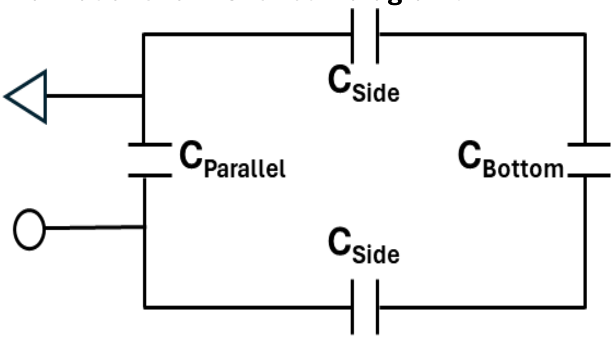

Capacitor values:

Coil Name	Cp$_{arallel}$(pF)	Cs$_{ides}$(pF)	C$_{Bottom}$(pF)
**Loop 1 - R1**	137	20	48
**Loop 1 – R2**	141	50.1	11.7
**Loop 1 – R3**	137	20.1	42.8
**Loop 2 - R1**	138	21.8	32.8
**Loop 2 – R2**	138	24.1	23.9
**Loop 2 – R3**	135	19	52.3
**Loop 3 - R1**	131	18.3	74.2
**Loop 3 – R2**	139	42	12.3
**Loop 3 – R3**	134	21.3	33.3
**Loop 4 - R1**	139	20.2	44.5
**Loop 4 – R2**	137	67.6	10.1
**Loop 4 – R3**	137	19.6	47.5
**Loop 5 - R1**	145	38.9	13.3
**Loop 5 – R2**	136	31	15.9
**Loop 5 – R3**	141	22.5	27.2
**Loop 6 - R1**	144	34.2	16
**Loop 6 – R2**	140	24.8	22.9
**Loop 6 – R3**	145	44.6	12.5

Single-row Overlapping Coil

Channel Numbering:

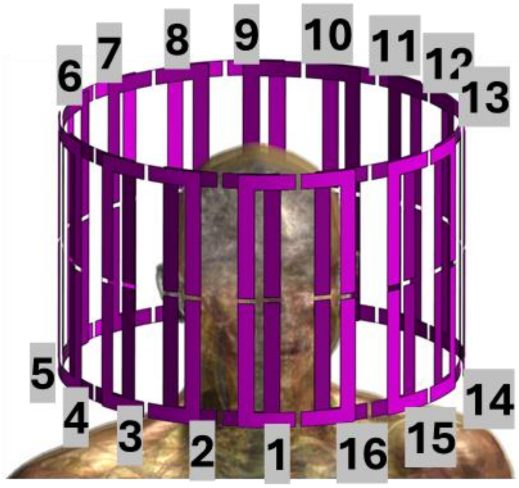

Individual channel circuit diagram:

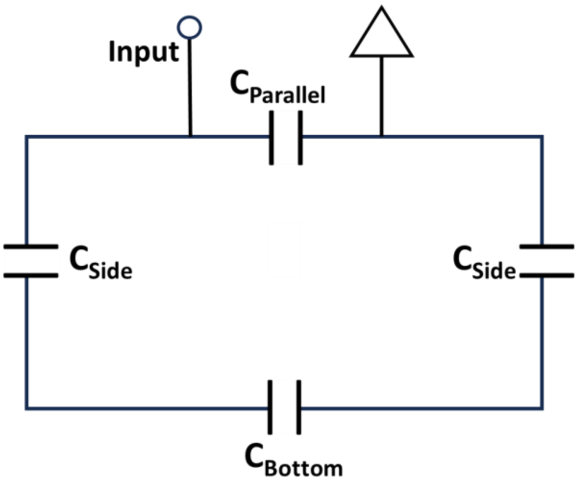

Capacitor values:

Coil Name	$Cp_{arallel}$(pF)	Cs_{ides}(pF)	C_{Bottom}(pF)
**Loop 1**	60	19.3	9.4
**Loop 2**	41	11.6	36.4
**Loop 3**	39.5	14.8	14.6
**Loop 4**	40	11.3	40.4
**Loop 5**	50	12	27.7
**Loop 6**	40	13.8	17.9
**Loop 7**	42	14.1	16
**Loop 8**	50	41.3	5.38
**Loop 9**	54.5	17.5	10.8
**Loop 10**	50	11.8	29.2
**Loop 11**	58	13	20.4
**Loop 12**	48	11.2	38.8
**Loop 13**	61	27.2	6.5
**Loop 14**	55	18.4	10.1
**Loop 15**	55	11.6	33.3
**Loop 16**	50	13.4	18.7

Double-row Overlapping Coil

Channel Numbering:

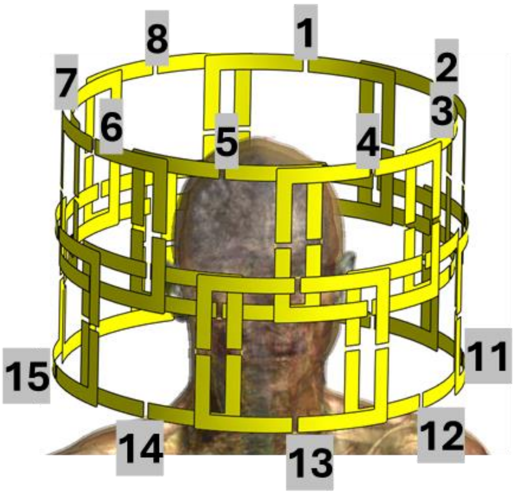

Individual channel circuit diagram:

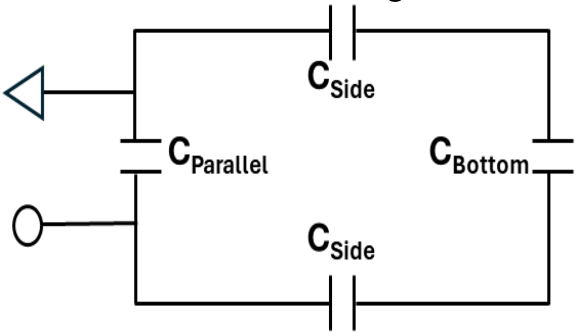

Capacitor values:

Coil Name	$C_{parallel}$(pF)	C_{sides}(pF)	C_{Bottom}(pF)
**Loop 1 - R1**	60	19.3	9.4
**Loop 2 - R1**	41	11.6	36.4
**Loop 3 - R1**	39.5	14.8	14.6
**Loop 4 - R1**	40	11.3	40.4
**Loop 5 - R1**	50	12	27.7
**Loop 6 - R1**	40	13.8	17.9
**Loop 7 - R1**	42	14.1	16
**Loop 8 - R1**	50	41.3	5.38
**Loop 1 - R2**	54.5	17.5	10.8
**Loop 2 - R2**	50	11.8	29.2
**Loop 3 - R2**	58	13	20.4
**Loop 4 - R2**	48	11.2	38.8
**Loop 5 - R2**	61	27.2	6.5
**Loop 6 - R2**	55	18.4	10.1
**Loop 7 - R2**	55	11.6	33.3
**Loop 8 – R2**	50	13.4	18.7

Triple-row Overlapping Coil

Channel Numbering:

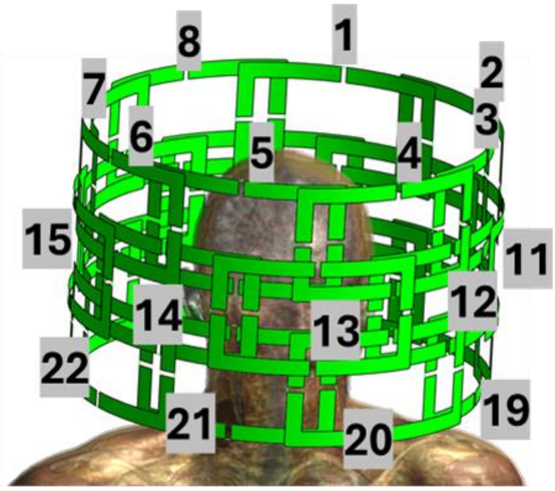

Individual channel circuit diagram:

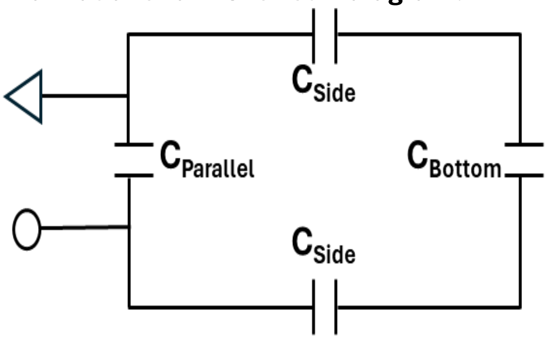

Capacitor values:

Coil Name	$C_{parallel}$(pF)	C_{Sides}(pF)	C_{Bottom}(pF)
**Loop 1 - R1**	38	14.9	36.6
**Loop 2 - R1**	40.4	32.6	9.31
**Loop 3 - R1**	41.8	23.4	12.9
**Loop 4 - R1**	45	30.9	9.85
**Loop 5 - R1**	38.8	15.5	31.6
**Loop 6 - R1**	42	18.7	19
**Loop 7 - R1**	42	25.4	11.5
**Loop 8 - R1**	40	14.3	49.7
**Loop 1 - R2**	40.7	20.8	14.6
**Loop 2 - R2**	34.7	14.7	36.5
**Loop 3 - R2**	41.9	18.4	18.3
**Loop 4 - R2**	35.4	15.5	29.1
**Loop 5 - R2**	50	27	10.2
**Loop 6 - R2**	45	33.2	9.12
**Loop 7 - R2**	42	20.8	15
**Loop 8 – R2**	36.1	14.8	35.2

**Loop 1 – R3**	43.5	44.7	7.54
**Loop 2 – R3**	40.4	27.2	11.2
**Loop 3 – R3**	40.1	18.7	19.3
**Loop 4 – R3**	40	19.9	17.2
**Loop 5 – R3**	40.2	21.9	14.3
**Loop 6 – R3**	40	15.6	32.8
**Loop 7 – R3**	39.5	23.7	12.7
**Loop 8 – R3**	42	22.2	14.2
